# Precise levels of nectin-3 are required for proper synapse formation in postnatal visual cortex

**DOI:** 10.1186/s13064-020-00150-w

**Published:** 2020-11-07

**Authors:** Johanna Tomorsky, Philip R. L. Parker, Chris Q. Doe, Cristopher M. Niell

**Affiliations:** 1 Institute of Neuroscience, University of Oregon, Eugene, OR 97403 USA; 2 Department of Biology, University of Oregon, Eugene, OR, 97403 USA; 3 Stanford University, 318 Campus Drive, Stanford, CA 94305 USA; 4 Institute of Molecular Biology, University of Oregon, Eugene, OR, 97403 USA; 5 Howard Hughes Medical Institute, University of Oregon, Eugene, OR, 97403 USA

**Keywords:** Visual cortex, Nectin-3, Postnatal development, Eye opening, Synapse formation, Dendritic spine density, Layer 2/3

## Abstract

**Background:**

Developing cortical neurons express a tightly choreographed sequence of cytoskeletal and transmembrane proteins to form and strengthen specific synaptic connections during circuit formation. Nectin-3 is a cell-adhesion molecule with previously described roles in synapse formation and maintenance. This protein and its binding partner, nectin-1, are selectively expressed in upper-layer neurons of mouse visual cortex, but their role in the development of cortical circuits is unknown.

**Methods:**

Here we block nectin-3 expression (via shRNA) or overexpress nectin-3 in developing layer 2/3 visual cortical neurons using *in utero* electroporation. We then assay dendritic spine densities at three developmental time points: eye opening (postnatal day (P)14), one week following eye opening after a period of heightened synaptogenesis (P21), and at the close of the critical period for ocular dominance plasticity (P35).

**Results:**

Knockdown of nectin-3 beginning at E15.5 or ~ P19 increased dendritic spine densities at P21 or P35, respectively. Conversely, overexpressing full length nectin-3 at E15.5 decreased dendritic spine densities when all ages were considered together. The effects of nectin-3 knockdown and overexpression on dendritic spine densities were most significant on proximal secondary apical dendrites. Interestingly, an even greater decrease in dendritic spine densities, particularly on basal dendrites at P21, was observed when we overexpressed nectin-3 lacking its afadin binding domain.

**Conclusion:**

These data collectively suggest that the proper levels and functioning of nectin-3 facilitate normal synapse formation after eye opening on apical and basal dendrites in layer 2/3 of visual cortex.

## Background

The development of mature brain circuits requires the selective formation, elimination, and strengthening of synaptic connections between neurons [1, 2]. This process requires the timed expression and spatial localization of proteins that are just beginning to be elucidated [3–5]. Many studies indicate that cell-adhesion molecules are involved in the formation and strengthening of synapses, though much is unknown about their specific roles in the maturation of various brain circuits [6–11]. The number and strength of synapses found on visual cortical neurons, inferred from dendritic spine density and shape, depend on visual experience and developmental stage [1, 12–19]. During the postnatal development of visual cortex, neurons experience an early increase in dendritic spine densities following eye opening [18, 20, 21]. This period of heightened synaptogenesis is followed by the critical period for ocular dominance plasticity (ODP, postnatal day (P)21 – P35), which is associated with experience-dependent synaptic refinement and pruning [2, 4, 19, 20, 22]. Dendritic spine remodeling provides a mechanism for synapses to alter their strength and number based on experience, e.g. through activity-dependent synaptic plasticity, and is a critical component of normal cortical development [12, 14–16, 23, 24]. Moreover, each cortical layer has a distinct timeline for the development of its unique visual response properties [25], likely a result of layer-specific synapse remodeling. Here, we sought to characterize the role of the cell-adhesion molecule nectin-3 in spine density profiles in developing layer 2/3 (L2/3) visual cortical neurons.

**Fig. 1:**
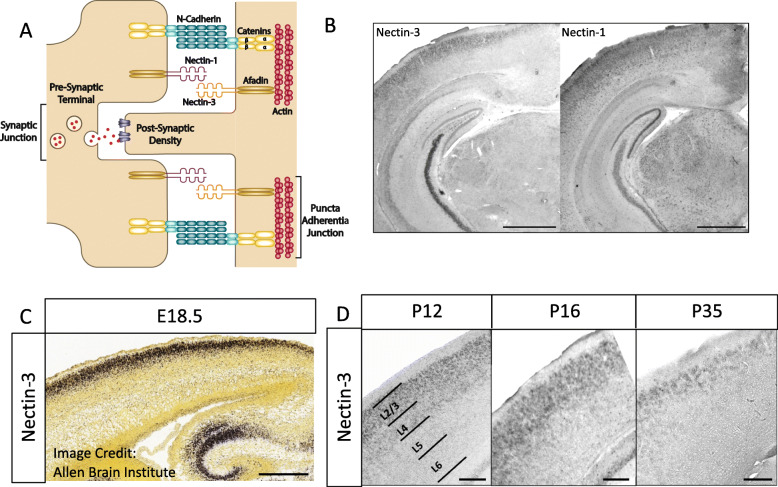
Model of synaptic nectin-3 and nectin-1 interactions and their expression in upper layers of cortex. **a** Nectin-1 and nectin-3 have been shown to bind at puncta adherentia junctions (PAJs) in hippocampus and interact with actin secondarily through their binding partner afadin [11, 26, 27]. Nectin and afadin have also been shown to associate with the N-cadherin/catenin complex at PAJs [11, 26–30]. Synaptic PAJs are stabilizing sites of adhesion between axons and dendrites and are distinct from synaptic junctions, which are the sites of neurotransmission [11, 27]. **b** In situ hybridizations (ISH) to nectin-3 and nectin-1 showing specific hippocampal and cortical expression patterns at P16. Both nectins demonstrate enriched expression in upper cortical layer neurons in V1 (2.5x objective, scale bar = 1 mm). **c** Nectin-3 expression is L2/3 specific as early as E18.5 (scale bar = 395 μm, image credit: Allen Brain Institute). **d** Nectin-3 expression is upper layer specific before (P12) and after (P16) eye opening and at the close of the critical period for ODP (P35) (scale bar = 200 μm)

The nectins are a group of immunoglobulin superfamily cell-adhesion molecules found to stabilize synapses at specialized structures called puncta adherentia junctions (PAJs) [26–29]. Trans-synaptic nectins and cadherins, together with their intracellular binding partners (afadin and catenins, respectively), help to form and remodel synapses over development (Fig. 1a) [11, 31]. For example, axonal (presynaptic) nectin-1 in dentate granule cells specifically binds to dendritic (postsynaptic) nectin-3 in CA3 principal neurons at the stratum lucidum in hippocampus to help guide and stabilize developing synaptic connections between these neurons (Fig. 1a) [11, 26]. Other studies have implicated nectins in a variety of biological and disease states, including long-term memory formation, stress, tauopathy, abnormal development, and intellectual disability, indicating that nectins may have different functions in the development, aging, and maintenance of various brain circuits [32–38]. While previous work suggests that nectin-1 and nectin-3 may be involved in both the formation and maturation (stabilization) of synapses in hippocampus, their function in postnatal cortical development is unknown [11].

Here we identify a role for nectin-3 in the development of visual cortical neurons by examining dendritic spine densities after manipulating neuronal nectin-3 expression in vivo. Mammalian cortex has a conserved laminar structure, and nectin-1 and nectin-3 both have distinct upper layer expression patterns in postnatal mouse cortex (Fig. 1b) [5, 32, 39]. This expression pattern suggests a potential role for nectin-3 in the development of L2/3-specific visual response properties after eye opening [25]. We assessed the dendritic spine densities of apical and basal dendrites on L2/3 visual cortical neurons at three developmental time points (P14, P21, and P35) after *in utero* electroporation of plasmid constructs expressing nectin-3 shRNA, full-length nectin-3, or nectin-3 lacking the four amino acid afadin-binding domain (Nec3^Δafadin^) [30, 40, 41]. Nectin-3 knockdown by shRNA beginning at embryonic day (E)15.5 or, using CaMKII-Cre transgenic mice [42], at ~P19, increased dendritic spine densities at P21 or P35, respectively. In contrast, overexpressing full-length nectin-3 produced an overall decrease in dendritic spine densities. These effects were most strongly associated with changes in proximal secondary apical dendrites. Interestingly, overexpressing Nec3^Δafadin^ produced an even greater decrease in dendritic spine densities than full-length nectin-3, particularly on basal dendrites at P21. These results collectively indicate that the neuronal regulation of synaptic nectin-3 may mediate both the formation and removal of synapses in L2/3 visual cortex during critical periods of development. This is the first study to examine the effect of nectin-3 on postnatal cortical development. While it was previously shown that blocking nectin-3 to nectin-1 binding in vitro can increase dendritic spine densities [11], here we uniquely identified spine density differences in vivo after manipulating nectin-3 levels in developing cortical neurons. It should also be noted that previous studies of adult hippocampal neurons observed decreased dendritic spine densities after nectin-3 knockdown in vivo [35, 36], indicating that the unique effect of nectin-3 observed here may be specific to cortical development.

## Methods

### In situ hybridization

The expression patterns of nectin-1 and nectin-3 were assayed by nonradioactive colorimetric RNA in situ hybridization, using solutions and probes as previously described [5, 43–45]. Briefly, animals were perfused and brains were fixed overnight in 4% PFA and then cryoprotected in a 30% sucrose solution. Brains were then cryosectioned to a thickness of 30 μm, mounted on Superfrost Plus slides (Fisherbrand), and stored at − 80 °C until use. 30 μm sections were brought to room temperature, washed in PBS and acetylated [45]. Slides were then pre-hybridized in hybridization solution in a humidity chamber for 2 h at 70 °C. The riboprobes to *Pvrl1* and *Pvrl3* (the genes encoding nectin-1 and nectin-3 proteins) were generated with dig-labeled nucleotides and SP6 RNA polymerase using probe sequences and protocols described by the Allen Brain Institute [39, 45, 46]. Riboprobes were diluted in hybridization solution to a concentration of 1–2 ng/μL, and slides were hybridized overnight at 70 °C with each probe. Slides were then washed (at 70 °C) and blocked for 1 h (at room temperature) before incubating overnight at 4 °C in anti-dig sheep Fab fragments conjugated to alkaline phosphatase (AP; Roche No. 11093274910) diluted 1:2500 in blocking solution. Slices were then washed at room temperature with MABT buffer and then AP staining buffer, after which 3.5 μL/mL NBT, 2.6 μL/mL BCIP, and 80 μL/mL levamisole in AP staining buffer was applied. The AP colorimetric reaction was observed closely as it developed for 3–48 h at 37 °C and was stopped by washing twice with PBS (0.1% Tween-20) and twice with deionized H_2_O. Slides were then dehydrated in graded ethanols and mounted using Permount Mounting Medium (Fisher).

### Design of Cre-dependent shRNA plasmids for *in utero* electroporation

Nectin-3 shRNA constructs were designed using previously published siRNA sequences [41]. For the double knockdown of nectin-1 and nectin-3 described in Figure S3 (Additional file 3), a nectin-1 shRNA construct was also designed using a previously untested siRNA sequence. 19 bp nectin-3, nectin-1, and scramble siRNA sequences were used to design the following shRNA oligos for cloning into a pSico vector [41, 47]:

Pvrl3 shRNA1 sense oligo: TGGCCGGATTCTTTAATTGATTCAAGAGTCAATTAAAGAATCCGGCCTTTTTTC.

Pvrl3 shRNA1 antisense oligo:

TCGAGAAAAAAGGCCGGATTCTTTAATTGACTCTTGAATCAATTAAAGAATCCGGCC A.

Pvrl3 shRNA2 sense oligo:

TGTTTATTGGCGTCAGATAATTCAAGAGATTATCTGACGCCAATAAACTTTTTTC.

Pvrl3 shRNA2 antisense oligo:

TCGAGAAAAAAGTTTATTGGCGTCAGATAATCTCTTGAATTATCTGACGCCAATAAACA.

Scr sense oligo:

TGCTACACTATCGAGCAATTTTCAAGAGAAATTGCTCGATAGTGTAGCTTTTTTC.

Scr antisense oligo:

TCGAGAAAAAAGCTACACTATCGAGCAATTTCTCTTGAAAATTGCTCGATAGTGTAGCA.

Pvrl1 shRNA1 sense oligo: TGCATTGTCAACTATCACCTTTCAAGAGAGGTGATAGTTGACAATGCTTTTTTC.

Pvrl1 shRNA1 antisense oligo:

TCGAGAAAAAAGCATTGTCAACTATCACCTCTCTTGAAAGGTGATAGTTGACAATGCA.

Cloning into pSico was modified from methods previously described [47]. Briefly, restriction enzymes XhoI and HpaI were used to digest the pSico vector (Addgene plasmid #11578). Digested vector RNA was then dephosphorylated using shrimp alkaline phosphatase (Roche) for 60 min at 37 °C to prevent re-ligation of the vector, followed by deactivation at 65 °C for 15 min. 1 μL of 100 μM sense and antisense shRNA oligos (synthesized by IDT) were then annealed and phosphorylated using T4 PNK (1 μL 10X T4 Ligation Buffer (NEB), 6.5 μL H2O, and 0.5 μL T4 PNK (NEB)). Annealing was performed in a thermocycler set to 37 °C for 30 min and then 95 °C for 5 min, followed by a ramp down to 25 °C at 5 °C/min. Vector and insert were then ligated with Quick Ligase (NEB) using manufacturers protocols. Ligated plasmid was then treated with Plasmid-Safe exonuclease (Lucigen), to prevent unwanted recombination products, and used to transform NEB Stable Competent *E. coli*. Positive colonies were grown in LB + amp., after which plasmid DNA was extracted (QIAprep Spin Miniprep Kit) and sequenced using the pSico sequencing primer: CAAACACAGTGCACACAACGC [47]. Plasmid DNA verified to contain the shRNA insert was then prepped for electroporation from 200 to 400 μL of cultured *E. coli* using a NucleoBond Midi or Maxi EF kit (Clontech) and eluted to a concentration of 5–10 μg/μL in TE.

### Expression constructs for *in utero* electroporation or HEK-cell transfection

Nectin-3 and nectin-1 overexpression constructs were created by modifying a pCag-iCre expression vector (Addgene plasmid #89573). Nectin-3 alpha was PCR amplified (KOD hot start DNA polymerase, Sigma-Aldrich) from a mouse brain cDNA library using the forward primer: GTTGAGGACACGCGCG and reverse primer: CTGTTAGACATACCACTCCCTCC. Similarly, nectin-1 was amplified from a mouse brain cDNA library using the forward primer: GTTTCTGGAGCGCGAG and reverse primer: GAAGTGGGCACAGACAGAG. Amplified DNA was run on a gel, and bands the length of nectin-3 (~ 1800 bp) or nectin-1 (~ 1500 bp) were cut from the gel and purified (QIAquick Gel Extraction Kit, Qiagen). Sequences were then amplified (KOD hot start DNA polymerase) using nested primers with and without a FLAG-tag and containing restriction sites for MluI and Not1. Primer sequences were as follows:

Nectin-3 F: TAAGCA-ACGCGT-GCCACC-ATGGCGCGGACCCCG.

Nectin-3 R: TGCTTA-GCGGCCGC-TTA-GACATACCACTCCCTCCTG.

Nectin-3 R Flag: TAAGCA-GCGGCCGC-TTA-CTTGTCGTCATCGTCTTTGTAGTC-GACATACCACTCCCTCCTG.

Nectin-1 F: TAAGCA-ACGCGT-GCCACC-ATGGCTCGGATGGGG.

Nectin-1 R: TGCTTA-GTTAACAA-TTA-CTTTACAGTTAGGGTGAG.

Dashes in forward primers demarcate 1) extra amino acids to improve restriction digest, 2) restriction sites, and 3) Kozak sequences before the start codon of nectin-3 or nectin-1 cDNA. Extra amino acids and restriction sites are also demarcated in reverse primers, in addition to the stop codon (TTA) and flag sequence (nectin-3 R Flag). DNA amplified using nested primers was gel purified and digested using the above-mentioned restriction enzymes. The pCAG-iCre plasmid was also digested with MluI and NotI to remove the iCre sequence from the vector. Digested vector and either nectin-3 or nectin-1 were then ligated, used to transform *E. coli*, and prepped for electroporation (NucleoBond Midi or Maxi EF kit, Clontech). Positive clones were sequenced using pCag F: GCAACGTTGCTGGTTATTGT, and Bglob-pA R: TTTTTGGCAGAGGGAAAAGAT sequencing primers.

A previously published construct expressing a truncated nectin-3 (lacking its 4 terminal amino acids, Nec3^Δ^^afadin^) was kindly gifted by Dr. Cristina Gil-Sanz and Dr. Ulrich Mueller [41]. FLEX-tdTomato constructs (plasmid #51509 and #51505) and the Cre-expression plasmid (plasmid #51904) were obtained from Addgene. All constructs were designed to express the nectin-3 alpha splice variant (with and without its four C-terminal amino acids).

### Western blot analysis of overexpression and shRNA constructs

**Fig. 2:**
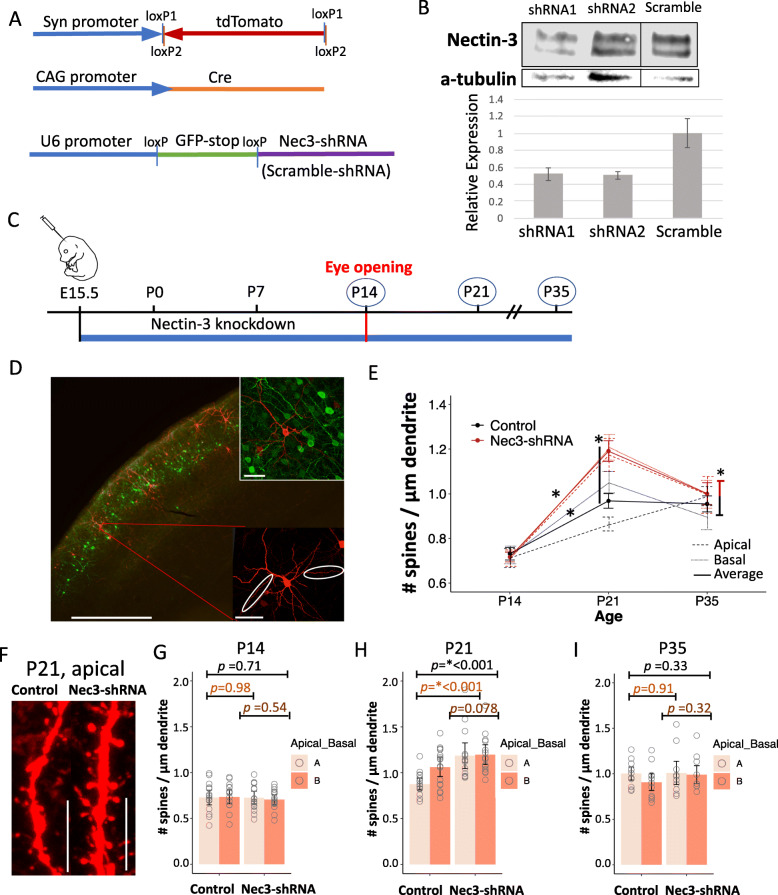
Nectin-3 knockdown at E15.5 increases dendritic spine density at P21. **a** Co-electroporated expression constructs. **b** Top: Western blot showing nectin-3 expression after co-transfection of HEK-293 cells with a nectin-3 expression plasmid and either nectin-3 or scramble shRNA constructs. Bottom: Nectin-3 expression/intensity was quantified and normalized to α-tubulin. Expression is shown relative to average nectin-3 expression from the scramble shRNA condition. **c** Electroporation/shRNA expression occurred at E15.5. Dendritic spine densities were analyzed at P14, P21, and P35. **d** Electroporated neurons at P21 in visual cortex (10x image, scale bar = 500 μm). Inset top: Cells expressing Cre-dependent tdTomato did not express floxed GFP-stop from pSico (40x image, scale bar = 50 μm). Inset bottom: Representative V1 neuron (40x image, scale bar = 50 μm), and select apical and basal dendrites. **e** Differences in dendritic spine densities between control and Nec3-shRNA neurons. Error bars denote standard errors of the mean. From left to right, asterisks denote significant differences for pooled apical and basal dendrites (solid line) between time points or between conditions at P21 (Additional file 5). **f** Representative apical dendrites from P21 Nec3-shRNA and control neurons (63x image, scale bar = 10 μm). **g** Nec3-shRNA and control dendritic spine densities at P14. Apical and basal dendritic spine densities are plotted. Error bars denote standard error of the mean. Each dot represents a single dendrite. One apical and one basal dendrite per cell were counted (Control: *N* = 15 cells, 30 dendrites, 5 animals; Nec3-shRNA: *N* = 16 cells, 32 dendrites, 6 animals). **h** Nec3-shRNA and control dendritic spine densities at P21. Significance is denoted by ‘*’ (Control: *N* = 17 cells, 34 dendrites, 5 animals; Nec3-shRNA: *N* = 14 cells, 28 dendrites, 5 animals). **i** Nec3-shRNA and control dendritic spine densities at P35 (Control: *N* = 11 cells, 22 dendrites, 3 animals; Nec3-shRNA: *N* = 10 cells, 20 dendrites, 5 animals)

The newly designed nectin-3 and nectin-1 overexpression constructs were transfected (Lipofectamine Reagent, Thermo Fisher Scientific) into HEK-293 cells using manufacturer’s protocols. A western blot was then run using 10 μg of protein extracted from transfected cells, as previously described [48]. When stained with an anti-nectin-3 antibody (Abcam Cat# ab63931, RRID:AB_1142394) western blot analysis showed greatly increased expression compared to endogenous HEK-293 expression (Fig. 5b). Similarly, the nectin-1 overexpression construct significantly increased nectin-1 expression when stained with an anti-nectin-1 antibody (Abcam Cat# ab66985, RRID:AB_2174031; Additional file 3: Figure S3). Western blots were also stained with an anti-alpha-tubulin antibody (Sigma-Aldrich Cat# T9026, RRID:AB_477593) to assess overall protein levels between conditions. To determine whether the newly designed shRNA constructs were effective, nectin-3 or nectin-1 overexpression and shRNA plasmids were co-transfected into HEK-293 cells. To establish the efficiency of co-transfection, lipofectamine was first used to co-transfect GFP and RFP expressing constructs into HEK-293 cells using manufacturers protocols. Using a 4:3 ratio of GFP to RFP constructs, nearly 100% co-transfection was observed (data not shown). pSico nectin-3-shRNA, nectin-1-shRNA, and scramble-shRNA constructs were altered before transfection to artificially replicate Cre-excision using site-directed mutagenesis (Q5 site directed mutagenesis kit: New England Biolabs). The GFP-stop sequence and one loxP site were removed from pSico constructs using the following primers: F: CGCATAACTTCGTATAGTATAAATTA, R: AATTACTTTACAGTTAGGGTGAG. The new shRNA plasmids did not require Cre for expression and were each co-transfected with nectin-3 (or nectin-1) overexpression constructs in HEK-293 cells at a 4:3 ratio. 10 μg of extracted protein was loaded onto a gel for western blot, and the blot was stained using nectin-3 (or nectin-1) and alpha-tubulin antibodies. After staining, nectin-3 expression/blot intensity was much higher than that observed for alpha-tubulin, making it difficult to observe shRNA knockdown by eye. To accurately assess relative protein levels between nectin-3 shRNA and scramble conditions, band intensities were quantified using Image Studio software, and the relative expression for each condition was obtained by normalizing nectin-3 intensity to alpha-tubulin intensity (technical replicates, *N* = 2, Fig. 2b). This analysis was repeated for nectin-1 shRNA, as shown in Additional file 3: Figure S3.

### *In utero* electroporation of plasmid DNA

All experimental protocols were approved by the University of Oregon Institutional Animal Care and Use Committees, in compliance with the National Institutes of Health guidelines for the care and use of experimental animals. *In utero* electroporation was performed at E15.5 to target L2/3 pyramidal neurons, as previously described [49, 50]. For shRNA knockdown using pSico, a solution of 2 μg/μL pSico-shRNA plasmid, 1.5 μg/μL FLEX-tdTomato plasmid, and 0.02 μg/μL Cre-expression plasmid was prepared in PBS (pH 7.2 for injections). In addition, 0.1% Fast Green dye was used to visualize plasmid DNA as it entered the ventricle with injection. For overexpression experiments using either full-length or truncated nectin-3 (Nec3^Δafadin^), a solution of 0.5 μg/μL of expression vector, 1μg/μL FLEX-tdTomato plasmid, and 0.01 μg/μL Cre-expression plasmid was prepared in PBS and 0.1% Fast Green.

Timed pregnancies were set up overnight between a hybrid strain of WT female mice (F1 cross of C57BL/6 J and 129S1/SvlmJ, Jax) and either the same strain of WT male mice or CaMKII-Cre homozygous transgenic male mice (Jax 005359). The day the plug was observed was designated embryonic day 0.5 (E0.5). 15.5-day pregnant mice were anesthetized with 2% isoflurane (0.8% O_2_) for the duration of the surgery. A small incision was made in the abdomen of pregnant females and the uterus was pulled out of the abdominal cavity. Approximately 1 μL of plasmid solution was injected through the uterus and scull into the lateral ventricle of E15.5 embryos [50]. Visual cortex was then targeted with an electrical pulse through tweezer-type electrodes using five, 45 V, 100 ms pulses at a 1 s interval. The uterus was then placed back in the abdominal cavity, the mouse was sutured, and allowed to recover. Animal health was monitored daily after surgery until pups were born (~ 4 days later). Electroporated mice were perfused as previously described [51] at P14, P21, or P35, and brains were prepared for immunohistochemistry.

### Immunohistochemistry

tdTomato fluorescence in electroporated neurons was amplified by immunohistochemistry (IHC) before imaging. Tissue preparation for IHC was performed as previously described [51]. Briefly, brains were perfused and then fixed overnight in 4% PFA (1x PBS), after which brains were immersed in 30% sucrose (1x PBS) for 24–48 h. Brains were then sliced on a vibratome to a thickness of 80 μm, placed in cryoprotectant solution (30% sucrose, 1% polyvinyl-pyrrolidone, 30% ethylene glycol in 0.1 M PB), and stored at − 20 °C. IHC was performed on free floating sections as previously described [51]. Briefly, sections were washed 3 × 10 min in a 0.7% glycine solution (in PBS) and then blocked for 1–3 h in PBST (0.3% Triton-X in PBS) containing 5% goat and 5% donkey serum. Slices were then transferred to a primary antibody solution containing 1.5 μL/mL of rabbit anti-RFP (Rockland Cat# 600–401-379, RRID:AB_2209751) and incubated overnight at 4 °C. The next day, slices were washed 1 × 10 min in PBST and then 3 × 10 min in PBS. Next, slices were transferred to a solution of 4 μL/mL of anti-rabbit Alexa Fluor 555 (Thermo Fisher Scientific Cat# A-21429, RRID:AB_2535850) secondary antibody in PBST and incubated at room temperature for 3 h. Slices were then washed for 10 min in PBST at room temperature, followed by an overnight wash in PBS at 4 °C. The next day, slices were washed an additional 2 × 10 min in PBS, treated with DAPI (4′, 6-diamidino-2-phenylindole), and mounted with VECTASHIELD mounting media (Vector Labs).

### Microscopy and spine counting

In situ hybridizations were imaged using an EC Plan-NEOFLUAR 5x/0.16 objective on a Zeiss Axio Imager.A2 wide field epifluorescence microscope having an X-Cite 120Q LED excitation lamp and a Zeiss AxioCam MRm 1.4-megapixel camera. ZEN lite imaging software (2012) was used to view images, and Adobe Photoshop CS6 was used for background removal and color processing of images.

Coronal sections of electroporated brain tissue were checked for fluorescent neurons in V1 using the Zeiss Axio Imager.A2 microscope and an EC Plan-NEOFLUAR 2.5x/0.085 objective. Immunohistochemistry was performed on sections confirmed to have electroporated cells. The location of electroporated neurons was determined using DAPI staining and a mouse brain atlas [52]. Neurons located V1 L2/3 were imaged on a ZeissLSM700 confocal microscope using Zen software. Images of secondary (at least one branch away from the soma) apical and basal dendrites were analyzed. Secondary apical dendrites directly extending laterally from the primary apical dendrite were preferentially selected (as opposed to apical tufts). High resolution images of dendrites for spine counting were taken using a Plan-Apochromat 63x/1.40 Oil DIC objective with 1.1–1.3x zoom, a speed of 8, averaging of 2, z-resolution of 0.3 μm, and variable laser intensities to capture dendritic spines. Spines in high resolution images of dendrites were counted manually using the open source FIJI image analysis software and the multipoint tool. Neurite lengths were measured using the ‘simple neurite tracer’ plugin. The mean length of dendrites analyzed was ~ 125 μm.

### Statistical analysis

In this study, apical and basal dendrites from a single cell were often analyzed and used as independent measurements of dendritic spine density. At least 9 cells per group, or 18 dendrites, were used to estimate average dendritic spine densities. The metadata for all experimental groups is included in Additional file 5 (Metadata sheet), including the number of animals and dendrites analyzed for all conditions and *p* values from individual t-tests examining differences between apical and basal dendrites. To account for possible cell-specific effects, we used a linear mixed model to analyze apical and basal dendrites together where ‘cell’ was included as a random effect. An ANOVA was then performed on modeled data using the Kenward-Roger method to determine degrees of freedom. Analyses were performed in R using the ‘lme4’ package. Individual comparisons between experimental (Nec3-shRNA, Nec3-OE, Nec3^Δafadin^) and control data were made at each age (P14, P21, and P35) using the following linear mixed model in R:

*lmer (Density ~ Condition + Dendrite type + (1|Cell))*

In this model, ‘condition’ and ‘dendrite type’ (apical or basal) are fixed effects, and ‘cell’ is included as a random effect. In addition, changes in dendritic spine densities between ages (P14 vs. P21 and P21 vs. P35) were analyzed for each condition using the following linear mixed model, where age is included as the first fixed effect:

*lmer (Density ~ Age + Dendrite type + (1|Cell))*

Modeled data was tested by ANOVA for differences in dendritic spine densities both between experimental groups (defined by condition or age) as well as between apical and basal dendrites (averaged over experimental groups). *P*-values from these comparisons are listed in Additional file 5.

**Fig. 3:**
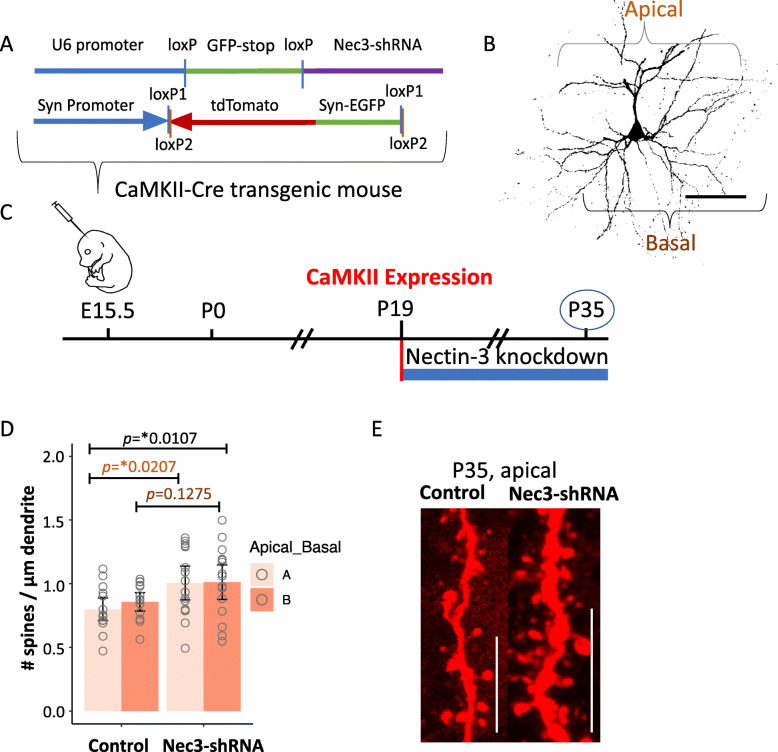
Nectin-3 knockdown at ~P19 increases dendritic spine density at P35. **a** A Cre-dependent shRNA construct to nectin-3 (or scramble shRNA construct) was co-electroporated with a Cre-dependent FLEX tdTomato plasmid (also expressing synaptophysin-EGFP) into developing CaMKII-Cre transgenic mice. **b** Both apical and basal dendrites on CaMKII-Cre/shRNA/tdTomato positive neurons were imaged at P35 (40x image, scale bar = 50 μm). **c** Mice were electroporated at E15.5 to target developing L2/3 neurons, but Cre recombination is not observed until ~P19 in the CaMKII-Cre mouse line [42]. For this experiment, neurons develop normally until nectin-3 knockdown at ~P19. Mice were sacrificed and neurons were imaged at P35. **d** Dendritic spine densities are significantly higher at P35 when nectin-3 is knocked down at ~P19 using a CaMKII-Cre mouse. Each dot represents a single dendrite. One apical and one basal dendrite per cell were counted. Significance is denoted by ‘*’ (Additional file 5, CKII-control: *N* = 14 cells, 28 dendrites, 5 animals; CKII-Nec3-shRNA: *N* = 16 cells, 32 dendrites, 6 animals). **e** Representative images of apical dendrites from control and Nec3-shRNA animals (63x image, scale bar = 10 μm)

Apical and basal dendrites were also analyzed separately to identify potential dendrite specific differences in spine densities related to nectin-3 manipulation or age. For this analysis, we used the ‘car’ package in R to perform an ANOVA where ‘density’ is tested against ‘condition’ (*aov (Density ~ Condition)*)*,* or age (*aov (Density ~ Age)*)*.* The data used in each comparison were first tested in R for normality and homoscedasticity using the Shapiro-Wilk normality test and Levene’s test for homogeneity of variance, respectively. If either test failed (*p* < 0.05), data was log2 transformed, after which all data passed both tests for normality and homoscedasticity (Additional file 5). Throughout this study, neurons electroporated with scramble shRNA were used as a control, except in Additional file 3: Figure S3, where neurons electroporated with either scramble shRNA (plus tdTomato and Cre), or tdTomato + Cre alone, were combined. All comparisons using a single nectin-3 manipulation and control data (between ages and between conditions at each age) were considered a family, and a Bonferroni corrected *p* value of 0.0055 was used to establish significant differences between groups (correction for a family of 9 comparisons). The experiment using a transgenic CaMKII-Cre mouse [42] for postnatal expression of Cre (Fig. 3) as well as the double nectin-3 and nectin-1 knockdown experiment described in Additional file 3: Figure S3 were both considered independent comparisons, and a *p* value of 0.05 was used as a cut-off for significance.

**Fig. 4:**
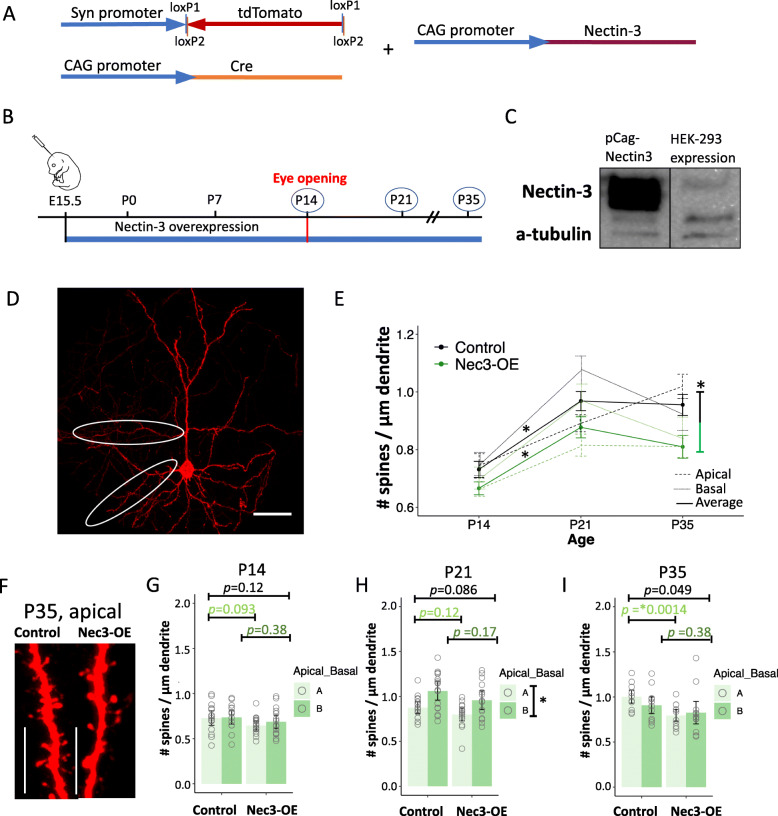
Nectin-3 overexpression beginning at E15.5 decreases dendritic spine density. **a** Co-electroporated expression constructs. **b** Electroporation/nectin-3 overexpression occurred at E15.5. Dendritic spine densities were analyzed at P14, P21, and P35. **c** Western blot analysis after transfection of HEK-293 cells with a nectin-3 overexpression vector. Greatly increased nectin-3 expression is observed relative to endogenous levels in HEK-293 cells. **d** Representative image of an electroporated neuron with select apical and basal dendrites circled (scale bar = 50 μm). **e** Nectin-3 overexpression produced an overall decrease in dendritic spine densities compared to control (scramble shRNA) neurons (pooled apical and basal dendrites, solid line). As with control neurons, spine densities significantly increased between P14 and P21, indicating spinogenesis was intact with nectin-3 overexpression. Significance is denoted by ‘*’, and *p* values are listed in Additional file 5. **f** Representative images of apical dendrites from control and Nec3-OE neurons at P35 (63x image, scale bar = 10 μm). **g** Nec3-OE and control dendritic spine densities at P14. Apical and basal dendritic spine densities are plotted. Error bars denote standard error of the mean. Each dot represents a single dendrite. One apical and one basal dendrite per cell were counted (Control: *N* = 15 cells, 30 dendrites, 5 animals; Nec3-OE: *N* = 16 cells, 32 dendrites, 6 animals). **h** Nec3-OE and control dendritic spine densities were not significantly different at P21 (Control: *N* = 17 cells, 34 dendrites, 5 animals; Nec3-OE: *N* = 14 cells, 28 dendrites, 5 animals). When Nec3-OE and control data were combined, apical dendritic spine densities were significantly lower than basal at P21. **i** Dendritic spine densities for Nec3-OE neurons trended lower than control neurons at P35 (pooled apical and basal dendrites). This result was significant when considering apical dendrites alone (Control: *N* = 11 cells, 22 dendrites, 3 animals; Nec3-OE: *N* = 12 cells, 24 dendrites, 3 animals)

**Fig. 5:**
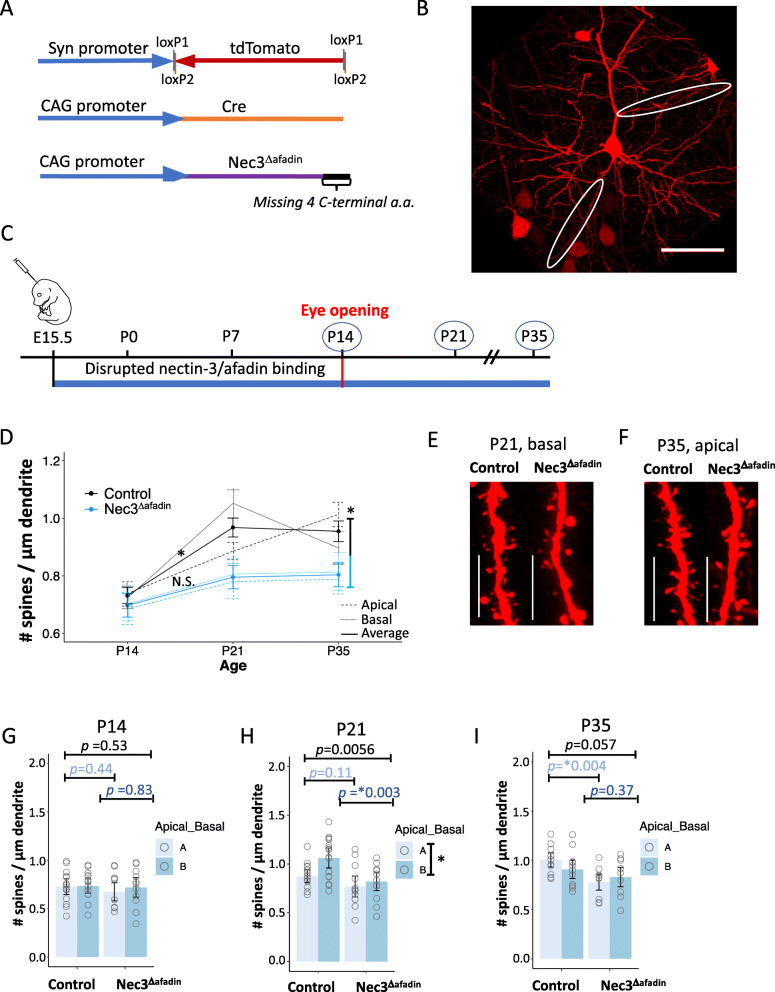
Overexpression of Nec3^Δafadin^ at E15.5 restricts spine formation after eye opening. **a** Co-electroporated expression constructs. **b** Representative image of an electroporated neuron with select apical and basal dendrites circled (scale bar = 50 μm). **c** Electroporation/dominant negative inhibition of nectin-3/afadin binding (Nec3^Δafadin^) occurred at E15.5. Dendritic spine densities were measured at P14, P21, or P35. **d** Nec3^Δafadin^ produced an overall decrease in dendritic spine densities compared to control neurons when all time points were considered (pooled apical and basal dendrites, solid line). In addition, dendritic spine densities did not significantly increase between P14 and P21. Significance is denoted by ‘*’, and *p* values are listed in Additional file 5. **e** Representative images of basal dendrites at P21 from Nec3^Δafadin^ and control neurons (63x image, scale bar = 10 μm). **f** Representative images of apical dendrites at P35 from Nec3^Δafadin^ and control neurons (63x image, scale bar = 10 μm). **g** Nec3^Δafadin^ and control dendritic spine densities at P14. Apical and basal dendritic spine densities are plotted along with error bars denoting the standard errors of the mean (Control: *N* = 15 cells, 30 dendrites, 5 animals; Nec3^Δafadin^, *N* = 16 cells, 32 dendrites, 6 animals). **h** Nec3^Δafadin^ and control dendritic spine densities at P21. Overall dendritic spine densities trended lower at P21 in Nec3^Δafadin^ neurons compared to control with a *p* value barely above the Bonferroni corrected *p* value of 0.0055. When basal dendrites were considered alone, a significant decrease in dendritic spine densities was observed (Control: *N* = 17 cells, 34 dendrites, 5 animals; Nec3^Δafadin^: *N* = 12 cells, 24 dendrites, 3 animals). **i** Nec3^Δafadin^ and control dendritic spine densities at P35. When apical dendrites were considered alone Nec3^Δafadin^ spine densities were significantly lower than control (Control: *N* = 11 cells, 22 dendrites, 3 animals; Nec3^Δafadin^: *N* = 9 cells, 18 dendrites, 3 animals)

Finally, we examined overall differences (combining all ages) in dendritic spine densities between control (scramble shRNA) and either Nec3-shRNA (Fig. 2), Nec3-OE (Fig. 4), or Nec3^Δafadin^ (Fig. 5) conditions. For the combined dataset including apical and basal dendrites, both ‘condition’ and ‘age’ were included as fixed effects in our model with a potential interaction:

lmer (Density ~ Condition*Age + Dendrite type + (1|Cell))

Differences between conditions were tested using an ANOVA (with Kenward-Roger approximation), and any significant interactions between ‘condition’ and ‘age’ were noted (Additional file 5). A similar analysis was performed on apical and basal dendrites separately, with condition and age considered as interacting effects (aov (Density ~ Condition*Age)). These overall comparisons were considered independent analyses, and a *p* value of 0.05 was considered significant. *p* values for comparisons between ages or conditions, as well as between apical and basal dendrite types, are listed in Additional file 5.

## Results

### Nectin-3 and nectin-1 are expressed in upper layers of visual cortex

Previous studies have shown a role for nectin-3 and its binding partner nectin-1 in the formation and maintenance of hippocampal synapses [11, 26, 27]. Mizoguchi et al. (2002) demonstrated using immunoelectron microscopy that nectin-1 and nectin-3 are distributed asymmetrically at hippocampal synapses, with nectin-1 located at the presynaptic side of mossy fiber terminals and nectin-3 located postsynaptically along the dendrites of pyramidal cells (Fig. 1a) [11]. In situ hybridization (ISH) revealed that nectin-3 and nectin-1 had specific expression patterns not only in hippocampus, where nectin-3 is enriched in CA3 and nectin-1 is enriched in the dentate gyrus, but also in cortex, where nectin-3 and nectin-1 are both enriched in upper layer cortical neurons (P16; Fig. 1b). In cortex, neurons in L4 send axons to L2/3, and L2/3 neurons are highly reciprocally connected within this layer [53, 54]. Our ISH data confirm data found at Allen Brain Atlas, showing upper layer specific expression of nectin-3 beginning at E18.5 and continuing through adulthood (Fig. 1c, d, Additional file 1: Figure S1) [39]. Nectin-1 first shows strong L2/3 expression at P4 and is upper layer enriched at P14 and P28 (Additional file 1: Figure S1). These expression patterns suggest that, similar to hippocampus, nectin-1 and nectin-3 may also interact during the development of cortical circuits.

### Nectin-3 knockdown at E15.5 increases dendritic spine density at P21

To knock down nectin-3 in developing neurons in vivo, we first designed new plasmid vectors allowing the Cre-dependent expression of nectin-3 short-hairpin RNA (shRNA) or scrambled control shRNA (Fig. 2a). We used previously published 19 bp siRNA sequences [41] to design our nectin-3 shRNA oligos and cloned these hairpins into the Cre-dependent pSico vector (Addgene plasmid #11578) [47]. pSico Cre-dependent shRNA constructs to nectin-3 (or scramble shRNA) contained loxP flanked GFP-stop sequences to prevent the expression of shRNA in the absence of Cre (Fig. 2a). shRNA constructs were co-electroporated with a Cre-expression plasmid and a Cre-dependent FLEX tdTomato construct (Fig. 2a). To test for knockdown of nectin-3, we co-transfected a nectin-3 expression plasmid with our new nectin-3 shRNA constructs (with the floxed GFP-Stop sequence removed, see Methods) into HEK-293 cells. Western blot analysis indicated that our newly designed nectin-3 shRNA constructs effectively reduced nectin-3 protein expression when normalized to a-tubulin and compared to cells co-transfected with a scrambled shRNA construct (technical replicates, *N* = 2, Fig. 2b). The nectin-3 expression construct used in this experiment led to very high nectin-3 expression in HEK-293 cells, making knockdown difficult to observe by eye (Fig. 2b, top). However, quantitative analysis using Image Studio software indicated consistently lower nectin-3 expression in cells where nectin-3 specific shRNA had been expressed (Fig. 2b, bottom). This is consistent with previously published data showing nectin-3 was knocked down in cultured cortical neurons using the same siRNA sequences [41].

To determine the effects of nectin-3 knockdown in vivo, we used *in utero* electroporation to introduce either or both nectin-3 shRNA1 and shRNA2 or scrambled shRNA (Fig. 2a, b) to developing L2/3 cortical neurons at E15.5 (Fig. 2b, c). pSico shRNA constructs were co-electroporated with a Cre-dependent tdTomato construct (at a 4:3 ratio) and a low concentration of a pCag-Cre plasmid (Fig. 2a, see methods for concentrations). Several previous studies have co-electroporated multiple plasmid constructs into developing mouse neurons with a high degree of efficiency [55–58]. One study found that when a 3:1 ratio of GFP to RFP expressing plasmids was co-electroporated, > 85% of GFP expressing cells also expressed RFP [55]. Since we were most concerned with the presence of our shRNA constructs, we used a higher concentration of these plasmids than any other construct (see methods). Using this paradigm, we are confident that the vast majority of cells expressing both the Cre-dependent tdTomato construct and the Cre construct (necessary to observe tdTomato) also expressed the more highly concentrated pSico shRNA expression plasmid. We further confirmed that cells expressing Cre-dependent tdTomato did not also express GFP, indicating the successful Cre excision of the GFP-stop sequence from the pSico construct (Fig. 2d, top inset). Since we demonstrated shRNA knockdown of nectin-3 when GFP-stop is excised from our constructs (allowing shRNA expression) in HEK cells, we have a high degree of confidence that nectin-3 knockdown was successful in tdTomato-positive cells (Fig. 2b).

After electroporation, animals were sacrificed at specific developmental time points, and we identified tdTomato expressing neurons in L2/3 of primary visual cortex (V1) for dendritic spine density analysis. Nectin-3 knockdown (or overexpression, Figs. 4 and 5) did not disrupt the migration of neurons to upper cortical layers (Fig. 2d, detailed migration analysis in Additional file 2: Figure S2). We selected two different dendrite types for dendritic spine density analysis: 1) secondary proximal apical dendrites extending sideways from the apical stalk, and 2) secondary basal dendrites at least one branch away from the soma (Fig. 2d, bottom inset). We assayed dendritic spine densities at three developmental time points: eye opening (P14), one week after eye opening (P21), and at the close of the critical period for ocular dominance plasticity (P35; Fig. 2c). This allowed us to assess nectin-3 function during critical periods in the development of visual cortex for synapse formation (P14 – P21) [18], as well as synaptic refinement (P21 – P35) [1, 22, 59].

Knocking down nectin-3 at E15.5 significantly impacted dendritic spine densities during postnatal development. When apical and basal dendrites were considered together, neurons electroporated with either scrambled shRNA (control) or nectin-3 shRNA constructs showed an increase in spine densities between P14 and P21, consistent with previous reports of synaptogenesis following eye opening in visual cortex (Control P14 – P21: *p* = 2.465e-05, Nec3-shRNA P14 – P21: *p* = 1.271e-10, Fig. 2e) [18]. Nectin-3 knockdown amplified this change, yielding significantly higher spine densities than control at P21 (Control – Nec3-shRNA: *p* = 0.00016, Fig. 2e, f, h). When analyzed separately, apical, but not basal, dendrites from Nec3-shRNA neurons showed a significant increase in spine densities relative to control neurons at P21 (Apical; Control – Nec3-shRNA: *p* = 9.633e-05, Basal; Control – Nec3-shRNA: *p* = 0.07893). We similarly found that knocking down both nectin-3 and nectin-1 increased dendritic spine densities relative to control neurons at P21, and that, again, apical dendrites were most significantly impacted (Control – Nec3 + Nec1-shRNA: *p* = 0.0014, Apical; Control – Nec3 + Nec1-shRNA: *p* = 0.0014, Basal; Control – Nec3 + Nec1-shRNA: *p* = 0.07063, Additional file 3: Figure S3). These results indicate that developing apical dendrites may be more sensitive to reduced nectin-3 expression than basal dendrites. Differences in dendritic spine densities between double knockdown and control neurons at P21 were present despite seeing no difference in dendrite complexity, as analyzed by Sholl analysis (Additional file 3: Figure S3f, g). By P35, the dendritic spine densities of Nec3-shRNA neurons were no longer significantly different from control neurons (Fig. 2i). Collectively, these results are consistent with a model where the extended knockdown of nectin-3 leads to a transient overproduction of weak spines after eye opening, which are readily pruned over the critical period for ocular dominance plasticity (P21 – P35) due to reduced stability.

### Nectin-3 knockdown at ~P19 increases dendritic spine density at P35

Early (E15.5) shRNA knockdown of nectin-3 yielded an overproduction of dendritic spines after eye opening, significantly increasing dendritic spine densities at P21 relative to control. On the other hand, dendritic spine densities between Nec3-shRNA and control neurons were not different at P35, indicating that the extra spines produced at P21 may have been removed over the critical period for ODP (P21 – P35). We next wanted to test whether the initial overproduction of spines at P21 after nectin-3 knockdown directly led to compensatory spine pruning in the following weeks. To accomplish this, we selectively knocked down nectin-3 near the start of the critical period for ODP and assessed dendritic spine densities at P35. For this experiment, we co-electroporated the same Cre-dependent Nec3-shRNA constructs (or control scramble shRNA) and a Cre-dependent tdTomato construct into transgenic CaMKII-Cre mice at E15.5 (Fig. 3a, c). In a previous study, CaMKII-Cre driven Cre/loxP recombination was first observed in cortex and hippocampus at P19 and expression was described as robust by P23 [42]. From this, we concluded that the knockdown of nectin-3 likely occurred in most cells around the start of the critical period for ocular dominance plasticity (P21). Both apical and basal dendritic spine densities were analyzed at P35, to allow time for full shRNA expression and knockdown of nectin-3 in L2/3 cortical neurons (Fig. 3b, c).

Unlike early (E15.5) shRNA knockdown of nectin-3, late nectin-3 knockdown (~P19) significantly increased dendritic spine densities at P35 relative to control (*p* = 0.0107, Fig. 3d, e). While both apical and basal dendritic spine densities appeared to increase with nectin-3 knockdown, when considered separately, only apical dendrites showed a statistically significant increase relative to control (*p* = 0.0207, Fig. 3d, e, Additional file 5). The critical period for ODP is typically associated with experience dependent spine pruning in V1, though different dendrite types have been shown to undergo different degrees of pruning [19, 60]. While our results could indicate that nectin-3 knockdown disrupts pruning over the critical period for ODP, the increased spine densities observed could equally be the result of increased spinogenesis. It is also possible that, as with early (E15.5) knockdown, the increased spine densities observed with late knockdown of nectin-3 may be transient. We did not take measurements of spine densities after P35, but other studies have found that nectin-3 knockdown in adult hippocampus decreases dendritic spine densities [35, 36]. Further studies are necessary to determine whether the developmental knockdown of nectin-3 has long-term effects in L2/3 cortical neurons.

### Nectin-3 overexpression at E15.5 decreases dendritic spine density

We next examined the effect of overexpressing nectin-3 in L2/3 cortical neurons over development. We generated a newly designed nectin-3 expression plasmid, which dramatically increased nectin-3 expression after transfection into HEK-293 cells (Fig. 4a, c). This nectin-3 overexpression construct was electroporated into developing L2/3 neurons at E15.5 (with a Cre-dependent tdTomato construct and a Cre-expression plasmid, Fig. 4a, b), and apical and basal dendritic spine densities were analyzed at P14, P21, and P35 (Fig. 4d). Dendritic spine densities on both control (scramble shRNA) and nectin-3 overexpression (Nec3-OE) neurons increased significantly between P14 and P21 (Control, P14 – P21: *p* = 2.47e-05; Nec3-OE, P14 – P21: *p* = 0.00017, Additional file 5). In addition, when all time points were considered together, Nec3-OE significantly decreased dendritic spine densities relative to control neurons (*p* = 0.0019, pooled apical and basal dendrites, Additional file 5, Fig. 4e). The greatest difference between Nec3-OE and control dendritic spine densities was observed at P35, and as with nectin-3 knockdown, spine densities were most significantly impacted on apical dendrites (pooled apical and basal, P35: *p* = 0.0492, apical dendrites alone, P35: *p* = 0.00136, Fig. 4i). Consistent with our shRNA experiments, these data further indicate that nectin-3 expression may restrict spinogenesis over development and/or facilitate pruning over the critical period for ODP (P21 – P35).

Our study indicates that the apical and basal dendrites we analyzed may experience different degrees of spinogenesis after eye opening. Individual t-tests were performed to determine whether apical and basal dendritic spine densities may differ within a single condition at a given age. Both control and Nec3-OE neurons demonstrated potential differences between apical and basal dendritic spine densities at P21 (Control, *p =* 0.0038; Nec3-OE, *p* = 0.029; Additional file 5, Metadata). When these datasets were combined in our statistical model (see methods), differences between apical and basal dendrites were highly significant (*p* = 4.14e-05, Additional file 5, Fig. 4g). This result suggests that the apical dendrites sampled in this study may not increase their spine densities after eye opening to the same degree as basal dendrites under the conditions of normal or above normal nectin-3 expression (Fig. 4e, h). It is important to note that this effect does not seem dependent on the overexpression of nectin-3, since no significant difference between Nec3-OE and control conditions was observed at P21 (Fig. 4g). Dendritic spine densities on apical dendrites continued to increase between P21 and P35 in the control condition alone. This indicates that, unlike other dendrite types [22, 61], proximal secondary apical dendrites (distinct from apical tufts) may be slower to increase dendritic spine densities after eye opening and may not undergo pruning over the critical period for ODP. Overexpression of nectin-3 most significantly decreased apical dendritic spine densities at P35, further indicating that the unique development of this dendrite type may be dependent on precise levels of nectin-3 expression. From this we conclude that dendrite type may also be an important consideration when evaluating developmental changes in dendritic spine densities following eye opening.

### Overexpressing nectin-3 lacking the afadin binding domain decreases dendritic spine density

Nectin proteins consist of a three extracellular Ig domains, a transmembrane fragment, and multiple intracellular C-terminal domains with distinct and independent functions [11, 30, 62–64]. Most nectins, including nectin-3, have a conserved motif of four amino acids at their cytoplasmic tail that binds the PDZ domain of afadin [11, 27, 30, 40]. Nectin-3/afadin binding is required for the interaction of nectin-3 with the actin cytoskeleton and the organization of PAJs in cooperation with N-cadherin (Fig. 1a) [11, 26–29, 31, 65]. Nectins can also recruit and activate c-Src leading to the downstream activation of Cdc42 and Rac, which facilitate the formation of filopodia and lamellipodia, respectively [63, 66–68]. The nectin-1 C-terminus, but not the afadin binding site, was necessary for the activation of Cdc42 and Rac [66]. The extracellular domain of nectin-3 has also been shown to act independently of its C-terminus to bind nectin-1, leading to nectin-1 intracellular signaling and cadherin recruitment [63, 66, 69]. It is unclear which nectin-3 domain may regulate dendritic spine formation during critical periods of postnatal development.

We next tested whether the decreased spine densities observed with nectin-3 overexpression required an interaction between nectin-3 and afadin. For this experiment, we used *in utero* electroporation to overexpress a nectin-3 protein lacking the four C-terminal amino acids required to bind afadin (Nec3^Δafadin^) [11, 27, 30, 40]. Developing L2/3 neurons were electroporated with Nec3^Δafadin^ (plus Cre and Cre-dependent tdTomato plasmids, Fig. 5a) at E15.5, and dendritic spine densities were assayed at P14, P21, and P35 (Fig. 5b, c). Note that the Nec3^Δafadin^ protein should still bind nectin-1 and activate Cdc42 and Rac signaling [63, 66–68]. Similar to the Nec3-OE condition, Nec3^Δafadin^ produced an overall decrease in dendritic spine densities relative to control neurons (scramble shRNA) when all time points were considered together (*p* = 0.00199, Fig. 5d). Also similar to Nec3-OE, Nec3^Δafadin^ neurons had decreased (trending) dendric spine densities relative to control at P35 (pooled apical and basal dendrites, P35: *p* = 0.057), which was significant when apical dendrites were considered independently (apical dendrites alone, P35: *p* = 0.004). From this we conclude that nectin-3 utilizes an afadin-independent pathway to restrict spine formation during this developmental period.

**Fig. 6:**
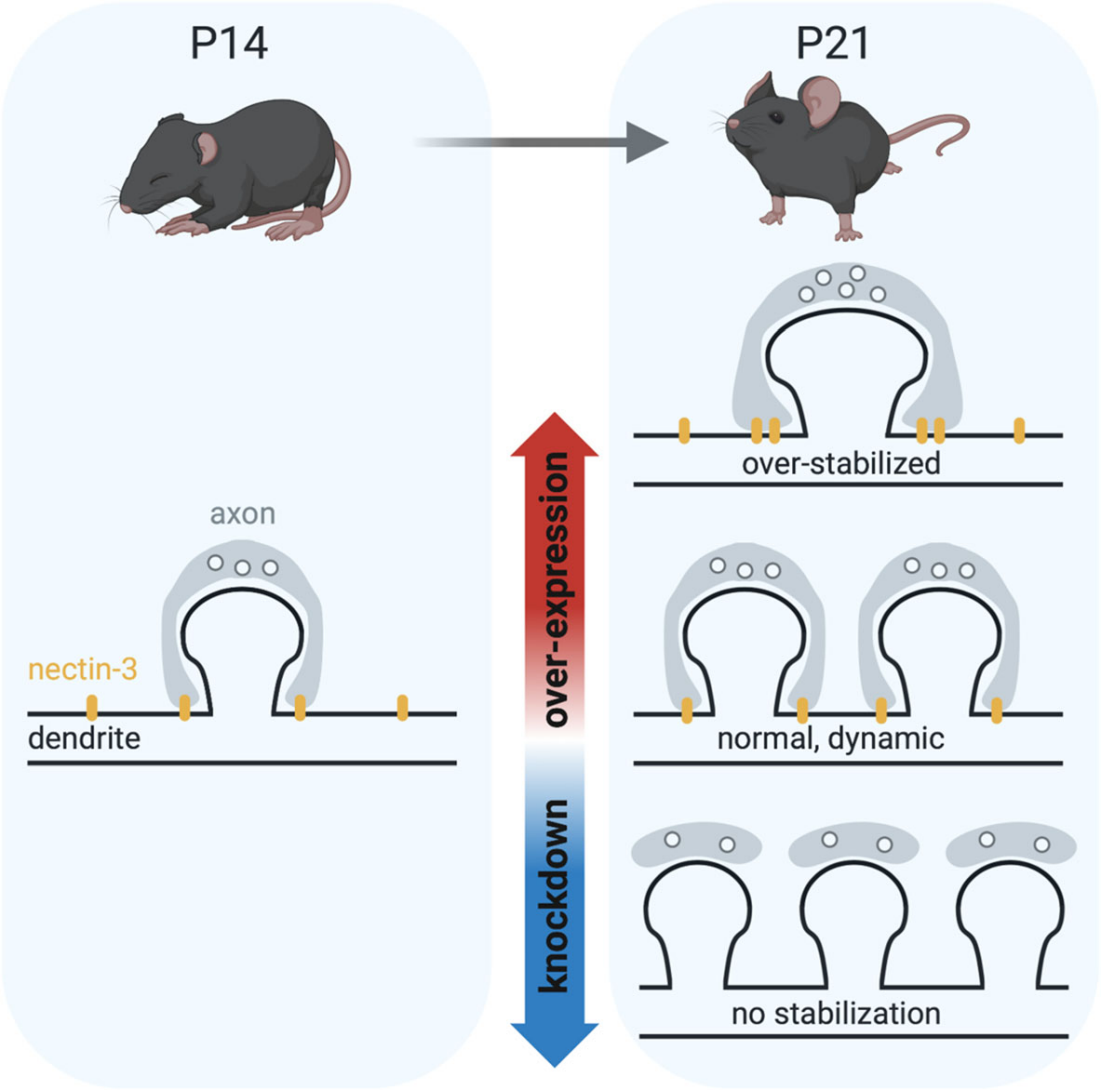
Hypothesized model summarizing results. After eye opening, L2/3 neurons experience an increase in dendritic spine densities. This period of spinogenesis may first require a weakening of synaptic adhesion at existing synapses. In this model, the over-stabilization of existing spines prevents new spine formation, while reduced synaptic adhesion facilitates new spine formation. It is further possible a similar but opposing mechanism facilitates pruning between P21 and P35; the strengthening of select spines by increased cell-adhesion facilitates the removal of neighboring weak spines. As a component of synaptic PAJs, nectin-3 may facilitate both the accumulation and removal of adhesion molecules at synaptic sites, perhaps through its association with afadin (created with BioRender.com)

Unlike Nec3-OE, the greatest difference between Nec3^Δafadin^ and control neurons was observed one week following eye opening, at P21 (pooled apical and basal dendrites, *p* = 0.0056). Interestingly, this effect appeared to be driven by basal dendrites, which were significantly decreased relative to control at P21 (*p* = 0.003). Furthermore, Nec3^Δafadin^ was the only condition where we did not observe a significant developmental increase in dendritic spine densities between P14 and P21 (Fig. 5d, Nec3^Δafadin^, P14 – P21: *p* = 0.1765). This result could suggest that, in the context of high nectin-3 expression, nectin-3-afadin binding may counteract the role of nectin-3 to suppress spine formation (see discussion). However, there are several possible explanations for why Nec3^Δafadin^ produced an enhanced phenotype at P21 relative to Nec3-OE, including differences in protein stability or expression levels between the two conditions. While further studies are necessary to fully understand the role of nectin-3-afadin binding in spinogenesis following eye opening, our results support an overall model whereby nectin-3 knockdown increases spine densities, and nectin-3 overexpression reduces spine densities, as described in Fig. 6 (representative images of dendrites and cells from all conditions and ages are shown in Additional file 4: Figure S4).

## Discussion

During the first 3 weeks following eye opening, the connectivity and function of mouse visual cortex changes rapidly to adapt to the onset of visually evoked neuronal activity [2, 18, 70, 71]. This reorganization of synaptic connections is thought to occur over two distinct critical periods, P14 –P21 (eye opening), and P21 – P35 (the critical period for ODP) [2, 4, 18–20, 22]. It is largely unknown how cell adhesion molecules, including the L2/3 enriched binding partners nectin-3 and nectin-1, may affect synapse formation or pruning during these critical periods in visual cortex development. Previous studies examining hippocampal neurons have identified different roles for nectin-3 in regulating dendritic spine densities depending on age and system (in vivo vs. in vitro) [11, 35, 36]. Two studies found dendritic spine densities decreased with nectin-3 knockdown in adult mouse hippocampus in vivo [35, 36]. On the other hand, blockade of nectin-1 to nectin-3 binding in developing hippocampal neurons in vitro increased dendritic spine densities and decreased spine size [11]. Consistent with the latter, here we show that nectin-3 may function to limit dendritic spine densities following eye opening in L2/3 visual cortex. These observations collectively indicate that nectin-3 may have a role in synapse formation, pruning, or maintenance that changes throughout development and aging.

To identify potential roles for nectin-3 in postnatal cortical development, we used the powerful technique, *in utero* electroporation, to knockdown (Nec3-shRNA) or overexpress (Nec3-OE) nectin-3 in developing L2/3 visual cortical neurons in vivo. We additionally overexpressed a nectin-3 lacking its afadin binding site (Nec3^Δafadin^), which should have acted as a dominant negative for nectin-3/afadin binding, overwhelming endogenous nectin-3 function [41, 72]. We then assayed dendritic spine densities on neurons with modified nectin-3 expression at critical ages in the development of visual cortex (P14, P21 and P35). Consistent with previous literature [18, 19, 21], we observed a significant increase in dendritic spine densities between P14 and P21 for all conditions except Nec3^Δafadin^. In control neurons, this increase in spine densities occurred rapidly on basal dendrites, but appeared more prolonged for apical dendrites, increasing to a lesser extent between P14 and P21, and continuing to increase between P21 and P35 (Figs. 2, 4 and 5). Nec3-shRNA increased dendritic spine densities at P21, indicating that this treatment may have ‘lifted the breaks’ on spine formation at this time. On the other hand, Nec3-OE and Nec3^Δafadin^ both yielded significant overall decreases in dendritic spine densities, which appeared most pronounced following periods of spine formation for each dendrite type (P21 for basal dendrites and P35 for apical dendrites, Figs. 4 and 5). Nec3^Δafadin^ was the only condition to prevent a significant increase in basal (or overall combined) dendritic spine densities between P14 and P21 (Fig. 5d). Taken together, these results indicate that nectin-3 may function to restrict dendritic spine formation after eye opening in visual cortex.

One hypothesis is that nectin-3 may act to restrict the homeostatic scaling of synaptic strength in L2/3 neurons following eye opening in V1. It was previously shown that the frequency of spontaneous miniature excitatory synaptic currents (mEPSCs, an indicator of synapse number) increases in upper layer cortical neurons after eye opening [18]. This occurs simultaneously with a decrease in mEPSC amplitude, an indicator of synaptic strength [18]. Since synapse number and visual input both increase after eye opening, a decrease in synapse strength may be necessary to prevent neuronal overexcitability (homeostatic scaling) [18, 73]. If, by extension, high synaptic strength is prohibitive to increases in synapse number, nectin-3 knockdown may facilitate increases in synapse number by decreasing synaptic strength. It has previously been suggested that decreased synaptic adhesion may increase synapse number either to compensate for reduced synaptic strength [11], or by allowing the redistribution of membrane components to sites where new synapses form [74]. Another possibility is that nectin-3 knockdown facilitates new spine formation by freeing up synaptic molecules which would otherwise be restricted by PAJs (Fig. 1). In this way, nectin-3 and other synaptic adhesion molecules may help neurons balance synapse strength and number following eye opening.

Relative to Nec3-OE, Nec3^Δafadin^ produced an even greater decrease in dendritic spine densities, particularly on basal dendrites at P21. While this result could be due differences in protein levels or stability between conditions, another possible explanation is that the function of nectin-3 to limit spine formation after eye opening may be counteracted by an interaction with afadin, and by extension, the actin cytoskeleton. Cytoskeletal components have previously been shown to both influence and be influenced by the cell-adhesion molecules they associate with [75], and nectin proteins appear to be no exception [31]. In immature (7 days in vitro (DIV)) developing cultured hippocampal neurons, actin depolymerization (latrunculin A treatment) prevented the localization of nectin-1 to synaptic sites and decreased the size of synaptophysin clusters (an indicator of reduced synaptic strength) [31]. In older neurons (21 DIV), actin depolymerization increased the density of synaptophysin clusters (an indicator of increased synapse number) and decreased the size of nectin-1 puncta at synaptic sites [31]. Accordingly, it is possible that cytoskeletal changes following eye opening [20] may also influence the position and function of nectin-3, perhaps facilitating its removal from synaptic sites through an association with afadin. Further studies are necessary to determine whether the redistribution of nectin-3 away from synaptic sites may have a role in homeostatic synaptic scaling and synapse formation after eye opening [18].

While eye opening is associated with an increase in synapse number and a decrease in synapse strength, the opposite is true during critical periods for experience-dependent synaptic plasticity (critical period for ODP in V1, P21–P35) [76, 77]. At these times, active synapses require a mechanism for selective strengthening while inactive synapses are weakened and pruned [76, 77]. This may be facilitated by the selective accumulation of synaptic adhesion molecules at active synapses and competitive removal from inactive synapses [7, 9, 17]. For example, it was previously demonstrated that competition between dendritic spines for N-cadherin, which is colocalized with nectins at synapses, may drive experience-dependent pruning in mouse somatosensory cortex [9, 11]. Here we show that when nectin-3 is knocked down acutely (~P19–P35) during the critical period for ODP, using CaMKII-Cre transgenic mice [42], spine densities are higher at P35 when compared to control neurons (Fig. 3). This indicates that nectin-3 may facilitate synapse pruning during the critical period for ODP, though it is possible that synapse formation was also affected by this manipulation. One hypothesis is that competition between spines for nectin-3 and its associated molecules may lead to the selective strengthening of active spines and weakening of inactive spines [9]. Several molecules associated with nectin-3 at PAJs, including N-cadherin, catenins, and afadin, have been shown to localize to synapses in response to neuronal activity (Fig. 1a) [7, 8, 78–83]. Interestingly, nectin-3 has been shown to recruit N-cadherin (C-terminal domain) to adherens junctions in an afadin-dependent manner [62]. Further experiments are necessary to determine whether nectin-3 may also experience activity-dependent synaptic localization or may influence the localization of associated PAJ molecules. In this way, nectin-3 could influence the activity-dependent refinement of synaptic connections between L2/3 cortical neurons during the critical period for ocular dominance plasticity in V1.

There are a number of challenges associated with using *in utero* electroporation to manipulate protein expression in developing neurons. While our experimental design provided a high degree of confidence that the Cre (and tdTomato) expressing cells we analyzed also expressed the more highly concentrated nectin-3 shRNA and overexpression constructs, plasmid concentration in a given electroporated neuron can vary, potentially leading to increased phenotypic variability from neuron to neuron within a single condition. In addition, there is high endogenous variability in spine densities between dendrites and cells in L2/3 of visual cortex. We focused on proximal apical and basal dendrites beginning one branch away from the soma (secondary dendrites) in order to limit the effect of this variability, but it was still difficult to obtain robust estimates of population differences. Previous studies of developing mouse or rat visual cortex have varied in their descriptions of dendritic spine densities over the critical period for ODP [19, 20, 22, 60], and different dendrite types on L3 pyramidal neurons have been shown to experience different degrees of pruning [19]. Our results indicate that basal and apical (proximal secondary) dendritic spine densities may experience different developmental trajectories after eye opening. For this reason, we analyzed apical and basal dendrites both separately and as a combined dataset, where we considered dendrite type (apical vs basal) as a fixed effect in our statistical models (see methods). Nectin-3 manipulation had slightly different effects on apical and basal dendritic spine densities, but overall trends were consistent between dendrite types. Despite the limitations described, we were able to consistently demonstrate increased spine densities with nectin-3 knockdown and decreased spine densities with nectin-3 overexpression.

In this study, we identify nectin-3 dependent changes to dendritic spine densities on L2/3 visual cortical neurons that become evident after eye opening and are dependent on time of knockdown and association with afadin. We hypothesize that nectin-3 may have roles in the formation and strengthening specific synapses; preventing runaway spine formation at P21 and facilitating the selective removal of inactive spines between P21 and P35. Further experiments are necessary to validate these hypothesized roles for nectin-3 in the development of L2/3 visual cortical neurons. Similar to the experiment performed by Bian et al. (2015), it would be informative to examine whether nectin-1 to nectin-3 binding at specific synapses in cultured neurons facilitates the competitive accumulation of cadherin/catenin complexes to those synapses and reduction at other synapses [9]. Electrophysiological recordings or calcium indicator imaging of neuronal activity could help determine whether nectin-3 expression facilitates the refinement of visual response properties (such as orientation selectivity, receptive field structure, or contextual modulation) in L2/3 cortical neurons, which would be expected if nectin-3 enables selective synaptic strengthening and pruning. Furthermore, examining nectin-3-dependent effects on spine density and synapse function after dark rearing could help address whether nectin-3 is functionally regulated by neuronal activity. In conclusion, our demonstration of a novel mechanism for regulating spine formation after eye opening should provide a foundation for future studies of the role of nectins in the assembly of cortical circuits.

## Conclusions

We conclude that the expression level of nectin-3 in layer 2/3 visual cortical neurons influences dendritic spine densities after eye opening. We suggest that tightly regulating nectin-3 at synapses may provide a mechanism for controlling the strength and number of dendritic spines on specific neurons during critical periods of cortical development.

## Supplementary information

**Additional file 1: Figure S1.** Compiled data from Allen Brain Institute (developingmouse.brain-map.org) showing the expression patterns of nectin-1 and nectin-3 throughout development. Nectin-3 displays specific expression in upper layers of cortex beginning at E18.5 and remains specific throughout development. Nectin-1 expression appears in upper cortical layers at P4 and is enriched in upper cortical upper layers at P14 and P28.

**Additional file 2: Figure S2.** Cells migrate normally to supragranular and granular layers after nectin-3 manipulation. **a** Representative images of electroporated cells from each condition showing migration to upper layers of cortex. The upper third of cortex was defined as supragranular/granular while the bottom two thirds was defined as subgranular. **b** Cells in supragranular/granular and subgranular layers of cortex were counted in cortical slices from animals electroporated with scramble shRNA (control), nectin-3 shRNA, or overexpression constructs (Nec3-OE and Nec3^Δafadin^). The percentage of supragranular/granular (red) or subgranular (blue) cells relative to total cells is shown. Cortical slices from animals at all ages (P14, P21, and P35) were included in this analysis (Control: *N* = 11 animals, Nec3-shRNA: *N* = 14 animals, Nec3-OE: *N* = 11 animals, Nec3^Δafadin^: *N* = 12 animals).

**Additional file 3: Figure S3.** Double knockdown of nectin-1 and nectin-3 increases spine densities without changing dendrite complexity at P21. **a** All neurons were electroporated with the Cre-dependent tdTomato and Cre expression constructs shown on the left. Double knockdown neurons were also electroporated with Cre-dependent nectin-1 shRNA and nectin-3 shRNA constructs, shown on the right. Two different controls were included and combined in this experiment: 1) neurons electroporated with only tdTomato and Cre expression constructs (left), and 2) neurons electroporated with tdTomato, Cre, and scramble shRNA expression constructs. **b** All animals were electroporated at E15.5 (initiating knockdown) and analyzed at P21. **c** Nectin-1 shRNA successfully knocks down nectin-1 in HEK-293 cells co-transfected with a nectin-1 expression construct and a nectin-1 shRNA construct. Nectin-1 expression/intensity was normalized to α-tubulin and is shown relative to nectin-1 expression when treated with scramble shRNA (analyzed in Image Studio). **d** Double knockdown of nectin-1 and nectin-3 increases dendritic spine densities relative to control (cells expressing tdTomato alone or tdTomato + scramble shRNA). Left: all double knockdown cells are shown relative to all control cells analyzed. A significant increase in the dendritic spine densities of double knockdown neurons is observed when all dendrites are considered together and when apical dendrites are considered independently (Control: *N* = 26 cells, 52 dendrites, 8 animals; Nec3 + Nec1-shRNA: *N* = 11 cells, 22 dendrites, 5 animals). Right: dendritic spine densities for the subset of control and double knockdown cells evaluated using Sholl analysis (Control: *N* = 8 cells, 16 dendrites, 4 animals; Nec3 + Nec1-shRNA: *N* = 9 cells, 18 dendrites, 5 animals). **e** Representative dendrites from double knockdown and control neurons. **f** Representative double knockdown neuron evaluated using Sholl analysis, which quantifies the number of dendrite crossings at successively larger radii away from the cell body. **g** Sholl analysis was performed using Imaris image analysis software. No differences were observed between Nec3 + Nec1-shRNA double knockdown neurons and control neurons.

**Additional file 4: Figure S4.** Representative images of dendrites and cells at each age and from each condition. **a** Images of dendrites and cells from experiments where scramble shRNA (control), Nectin-3 shRNA, or overexpression vectors (Nec3-OE, and Nec3^Δafadin^) were electroporated at E15.5. **b** Representative images of dendrites and cells at the ages examined in this study (P14, P21, and P35).

**Additional file 5. **Summary of all statistical analyses and *p* values for comparisons between condition, ages, and dendrite type. The type of analysis as well as the Bonferroni critical value used to establish significance, are indicated. Whether the data was first transformed to correct for non-normality or heteroskedasticity is also indicated, and final *p* values for Shapiro and Levine tests (after any necessary data transformations) are shown. An additional sheet labeled ‘Metadata’ documents the number of animals and dendrites sampled for each condition. This sheet also includes *p* values describing individual t-test comparisons of apical and basal dendrites for each condition.

## Data Availability

All data and reagents are available upon request.

## Abbreviations

PAJ: Puncta Adherentia Junction
Nec3-shRNA: Nectin-3 short hairpin RNA
Nec3-OE: Nectin-3 overexpression
Nec3^Δafadin^: Nectin-3 missing the afadin binding site
siRNA: small interfering RNA
V1: Primary visual cortex
L2/3: Layer 2/3
P14: Postnatal day 14
ODP: Ocular dominance plasticity
IHC: Immunohistochemistry
