# Supplementary figures and images for: Precise levels of nectin-3 are required for proper synapse formation in postnatal visual cortex
Source: Neural Dev. 2020 Nov 7;15:13. doi: 10.1186/s13064-020-00150-w (PMC7648993; doi:10.1186/s13064-020-00150-w)

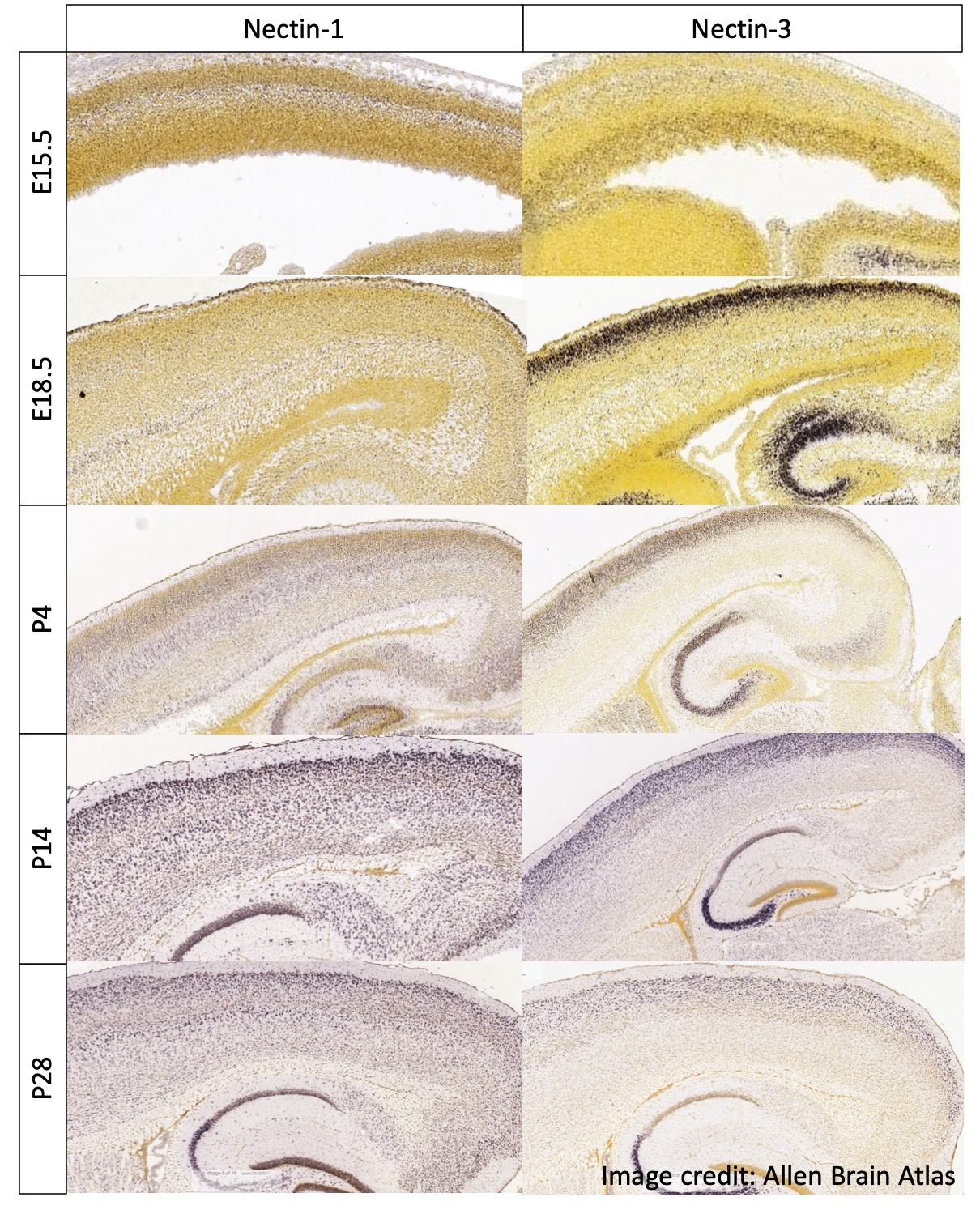

Supplement: Supplementary file 1 — Additional file 1: Figure S1. Compiled data from Allen Brain Institute (developingmouse.brain-map.org) showing the expression patterns of nectin-1 and nectin-3 throughout development. Nectin-3 displays specific expression in upper layers of cortex beginning at E18.5 and remains specific throughout development. Nectin-1 expression appears in upper cortical layers at P4 and is enriched in upper cortical upper layers at P14 and P28. [file 13064_2020_150_MOESM1_ESM.jpg]

A

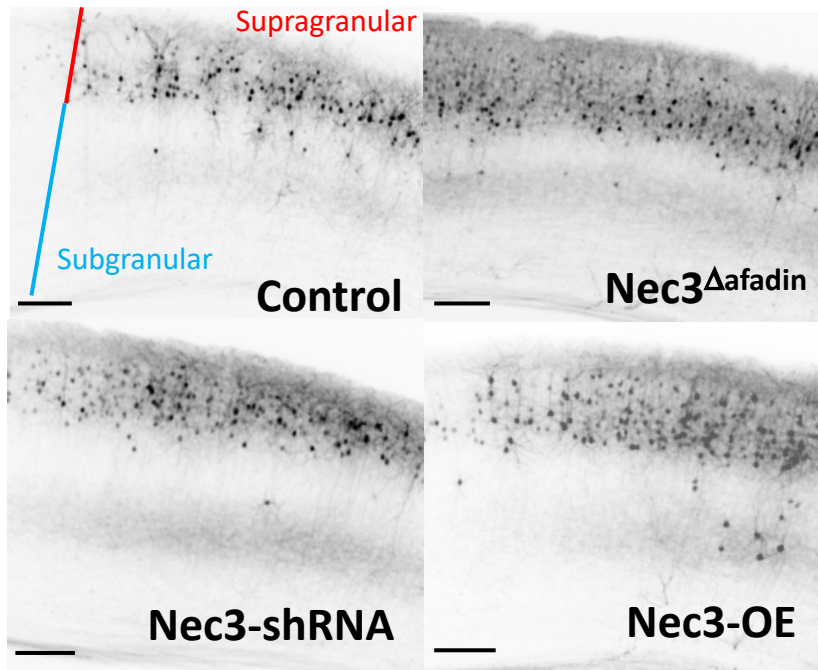

B

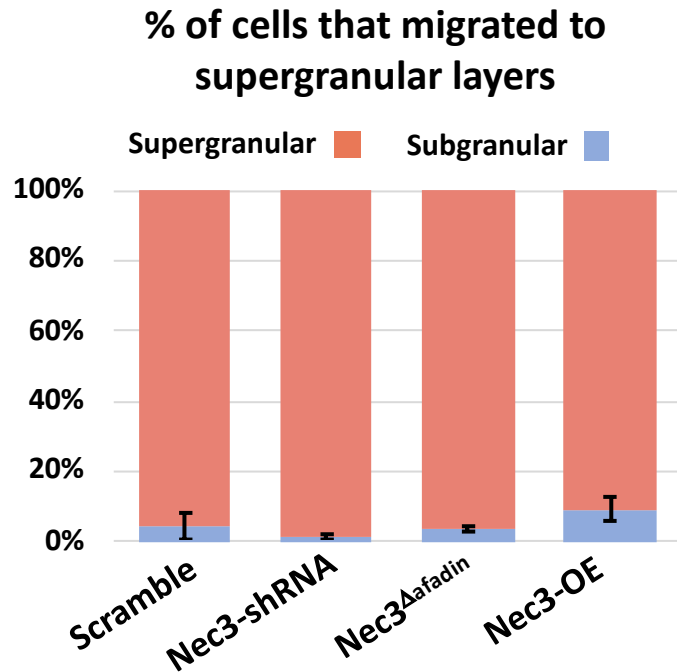

Supplement: Supplementary file 2 — Additional file 2: Figure S2. Cells migrate normally to supragranular and granular layers after nectin-3 manipulation. a Representative images of electroporated cells from each condition showing migration to upper layers of cortex. The upper third of cortex was defined as supragranular/granular while the bottom two thirds was defined as subgranular. b Cells in supragranular/granular and subgranular layers of cortex were counted in cortical slices from animals electroporated with scramble shRNA (control), nectin-3 shRNA, or overexpression constructs (Nec3-OE and Nec3Δafadin). The percentage of supragranular/granular (red) or subgranular (blue) cells relative to total cells is shown. Cortical slices from animals at all ages (P14, P21, and P35) were included in this analysis (Control: N = 11 animals, Nec3-shRNA: N = 14 animals, Nec3-OE: N = 11 animals, Nec3Δafadin: N = 12 animals). [file 13064_2020_150_MOESM2_ESM.pdf]

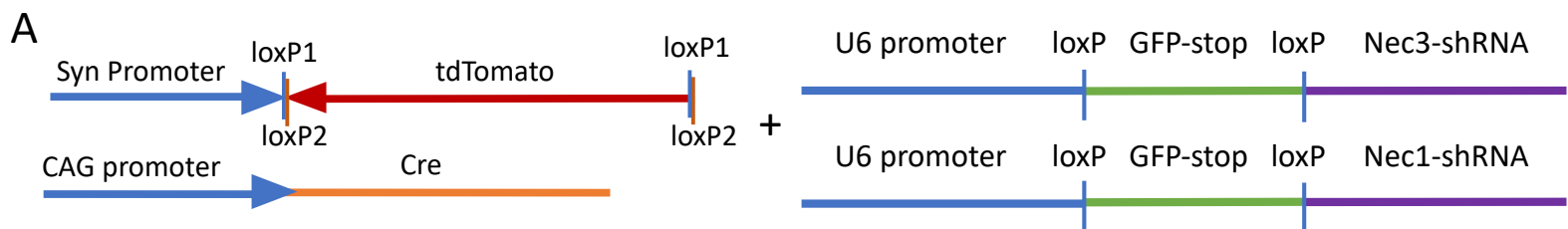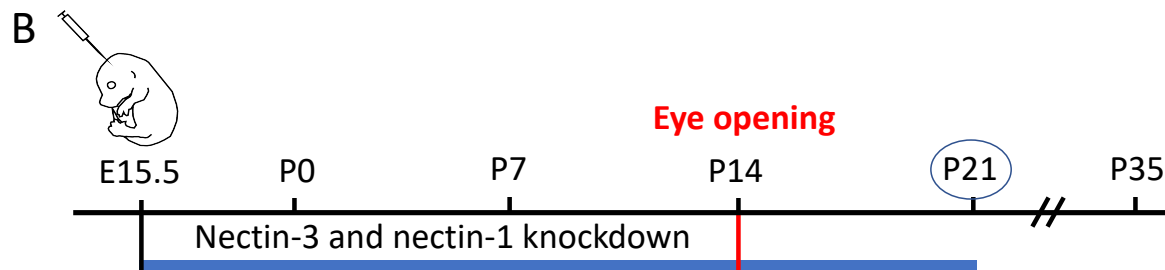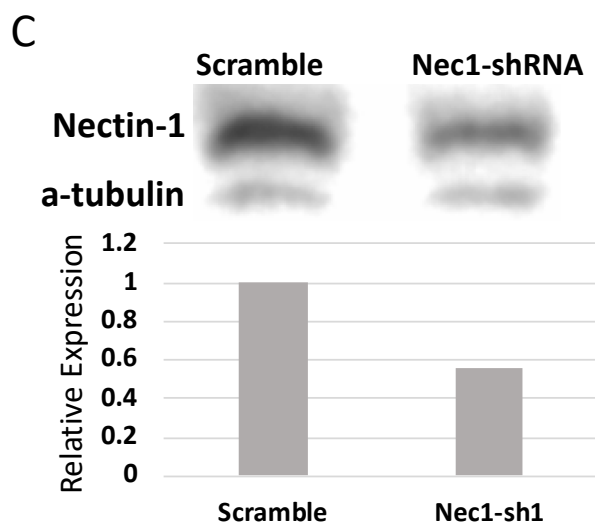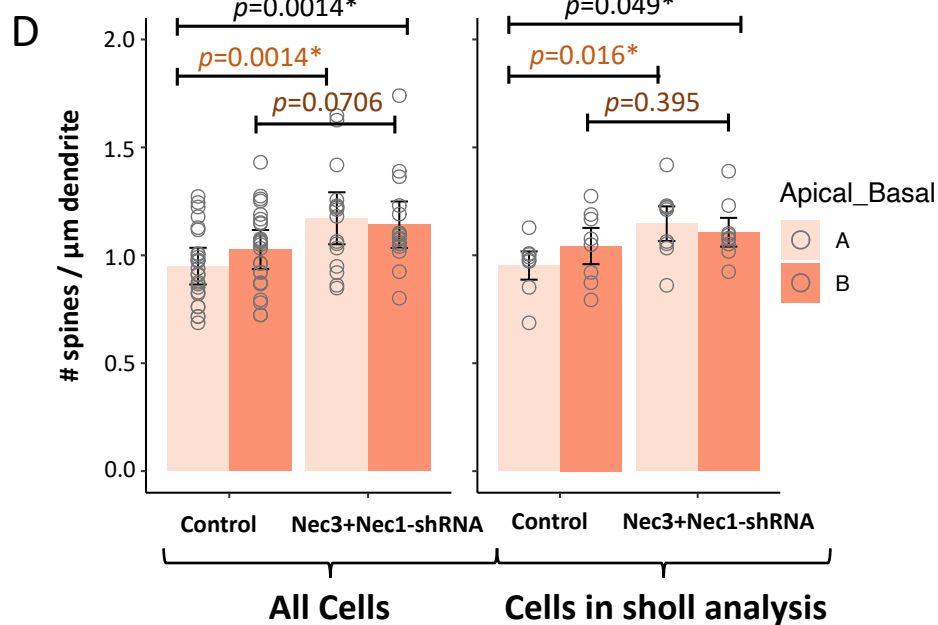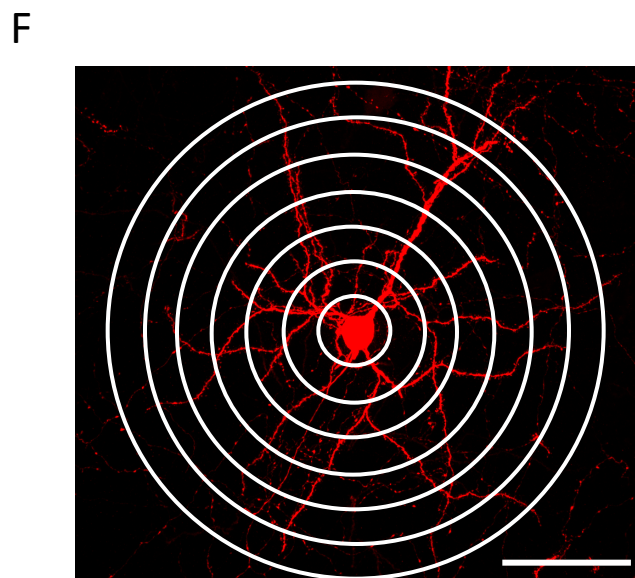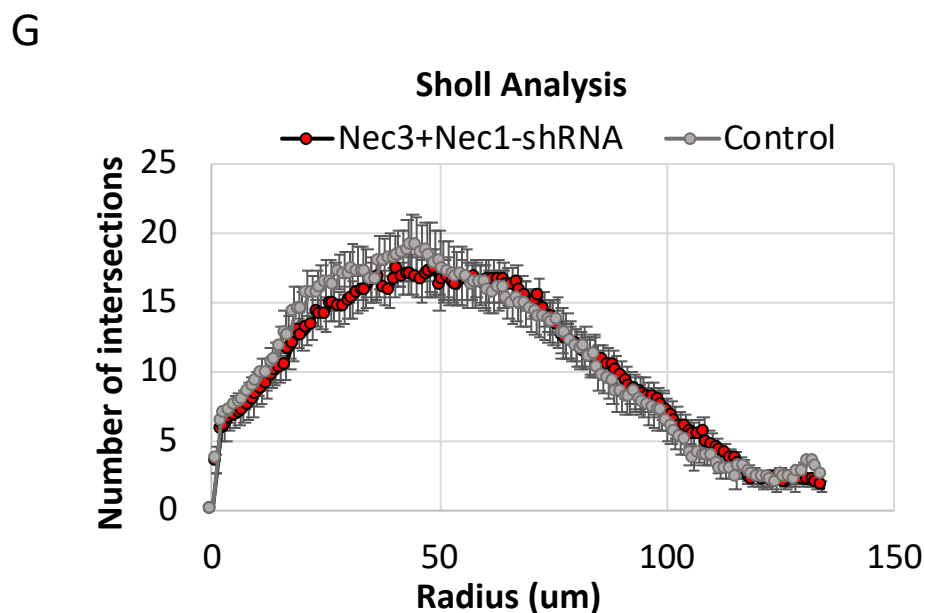

Supplement: Supplementary file 3 — Additional file 3: Figure S3. Double knockdown of nectin-1 and nectin-3 increases spine densities without changing dendrite complexity at P21. a All neurons were electroporated with the Cre-dependent tdTomato and Cre expression constructs shown on the left. Double knockdown neurons were also electroporated with Cre-dependent nectin-1 shRNA and nectin-3 shRNA constructs, shown on the right. Two different controls were included and combined in this experiment: 1) neurons electroporated with only tdTomato and Cre expression constructs (left), and 2) neurons electroporated with tdTomato, Cre, and scramble shRNA expression constructs. b All animals were electroporated at E15.5 (initiating knockdown) and analyzed at P21. c Nectin-1 shRNA successfully knocks down nectin-1 in HEK-293 cells co-transfected with a nectin-1 expression construct and a nectin-1 shRNA construct. Nectin-1 expression/intensity was normalized to α-tubulin and is shown relative to nectin-1 expression when treated with scramble shRNA (analyzed in Image Studio). d Double knockdown of nectin-1 and nectin-3 increases dendritic spine densities relative to control (cells expressing tdTomato alone or tdTomato + scramble shRNA). Left: all double knockdown cells are shown relative to all control cells analyzed. A significant increase in the dendritic spine densities of double knockdown neurons is observed when all dendrites are considered together and when apical dendrites are considered independently (Control: N = 26 cells, 52 dendrites, 8 animals; Nec3 + Nec1-shRNA: N = 11 cells, 22 dendrites, 5 animals). Right: dendritic spine densities for the subset of control and double knockdown cells evaluated using Sholl analysis (Control: N = 8 cells, 16 dendrites, 4 animals; Nec3 + Nec1-shRNA: N = 9 cells, 18 dendrites, 5 animals). e Representative dendrites from double knockdown and control neurons. f Representative double knockdown neuron evaluated using Sholl analysis, which quantifies the number of dendrite cros [file 13064_2020_150_MOESM3_ESM.pdf]

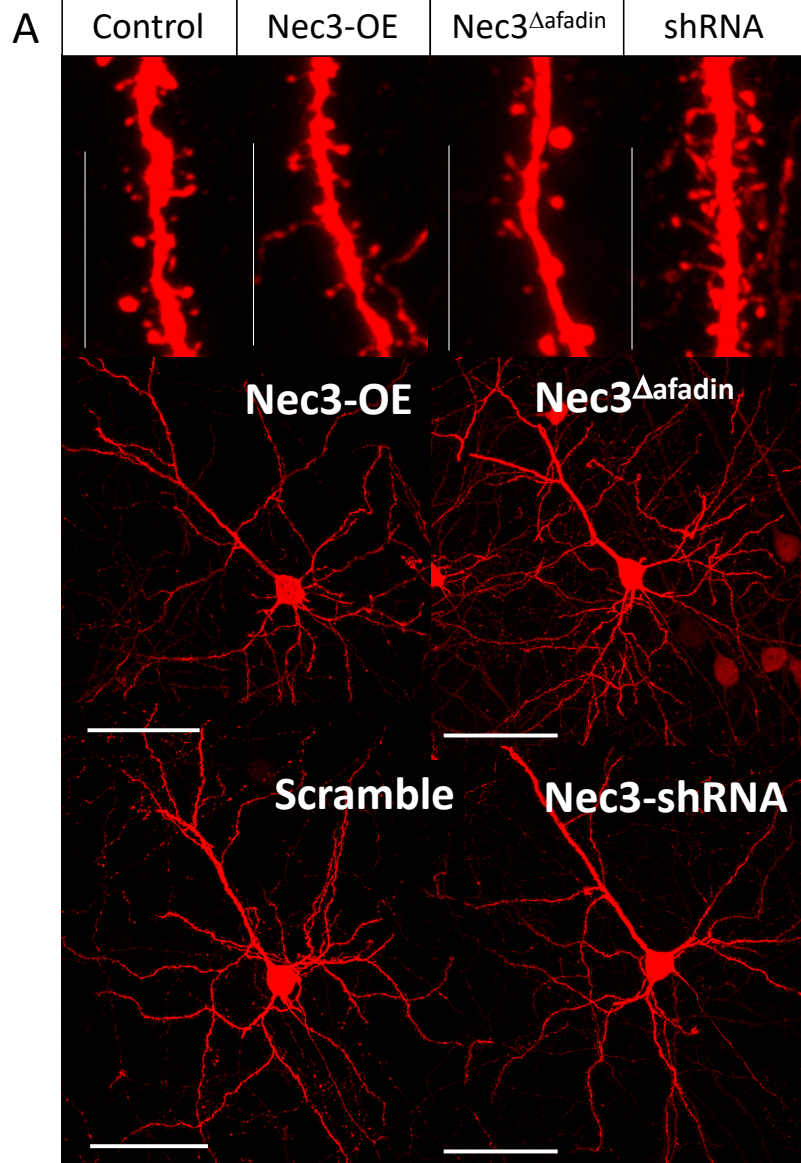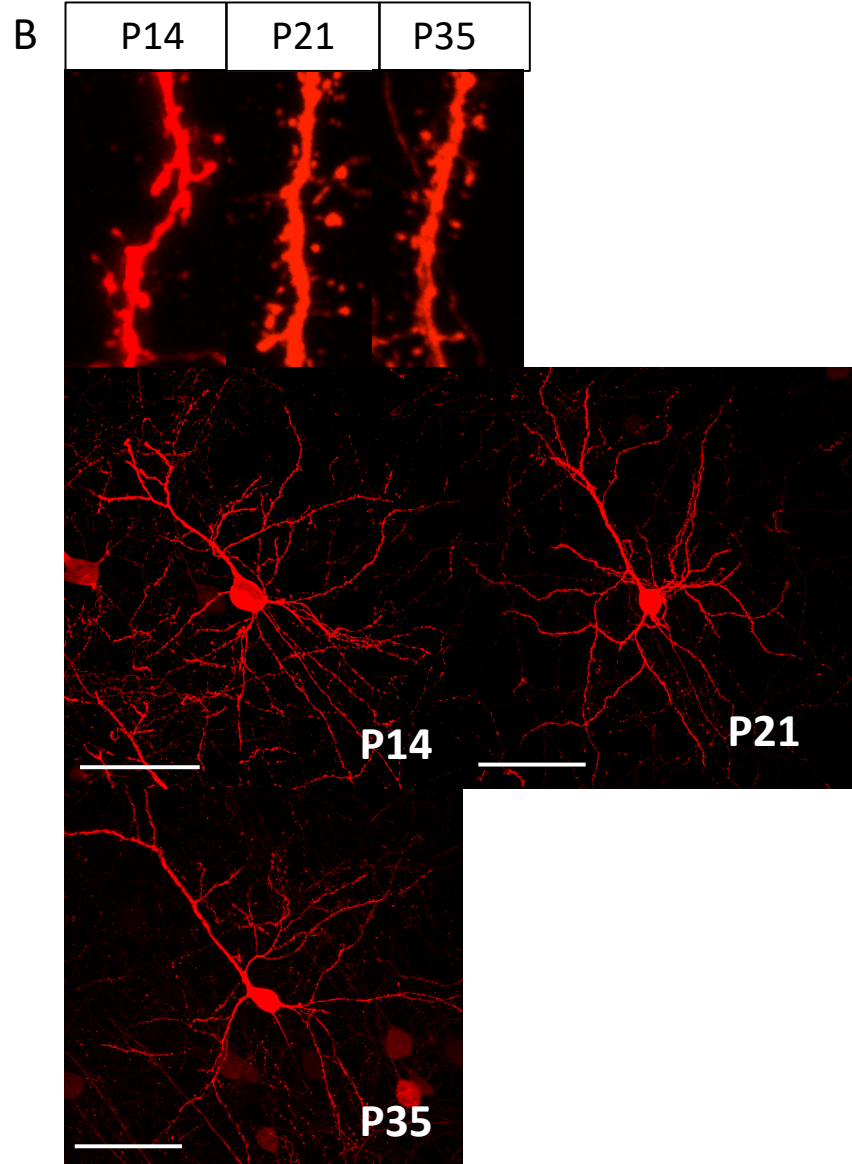

Supplement: Supplementary file 4 — Additional file 4: Figure S4. Representative images of dendrites and cells at each age and from each condition. a Images of dendrites and cells from experiments where scramble shRNA (control), Nectin-3 shRNA, or overexpression vectors (Nec3-OE, and Nec3Δafadin) were electroporated at E15.5. b Representative images of dendrites and cells at the ages examined in this study (P14, P21, and P35). [file 13064_2020_150_MOESM4_ESM.pdf]
